# The efficiency and safety of high-dose vitamin C in patients with COVID-19: a retrospective cohort study

**DOI:** 10.18632/aging.202557

**Published:** 2021-02-26

**Authors:** Dengfeng Gao, Min Xu, Gang Wang, Jianrui Lv, Xiaorong Ma, Yonghong Guo, Dexin Zhang, Huiyun Yang, Wei Jiang, Fuxue Deng, Guozhi Xia, Ziwei Lu, Lv Lv, Shouping Gong

**Affiliations:** 1 Department of Cardiology, The Second Affiliated Hospital, Xi’an Jiaotong University, Xi’an, Shaanxi, P.R. China; 2 Department of Critical Care, The Second Affiliated Hospital, Xi’an Jiaotong University, Xi’an, Shaanxi, P.R. China; 3 Department of Anesthesiology, The Second Affiliated Hospital, Xi’an Jiaotong University, Xi’an, Shaanxi, P.R. China; 4 Department of Hematology, The Second Affiliated Hospital, Xi’an Jiaotong University, Xi’an, Shaanxi, P.R. China; 5 Department of Infectious Diseases, The Second Affiliated Hospital, Xi’an Jiaotong University, Xi’an, Shaanxi, P.R. China; 6 Department of Respiratory and Critical Care, The Second Affiliated Hospital, Xi’an Jiaotong University, Xi’an, Shaanxi, P.R. China; 7 Department of Nursing, The Second Affiliated Hospital, Xi’an Jiaotong University, Xi’an, Shaanxi, P.R. China; 8 Department of Neurosurgery, The Second Affiliated Hospital, Xi’an Jiaotong University, Xi’an, Shaanxi, P.R. China

**Keywords:** coronavirus disease 2019, ascorbic acid, mortality, safety

## Abstract

Background: The inflammatory reaction is the main cause of acute respiratory distress syndrome and multiple organ failure in patients with Coronavirus disease 2019, especially those with severe and critical illness. Several studies suggested that high-dose vitamin C reduced inflammatory reaction associated with sepsis and acute respiratory distress syndrome. This study aimed to determine the efficacy and safety of high-dose vitamin C in Coronavirus disease 2019.

Methods: We included 76 patients with Coronavirus disease 2019, classified into the high-dose vitamin C group (loading dose of 6g intravenous infusion per 12 hr on the first day, and 6g once for the following 4 days, n=46) and the standard therapy group (standard therapy alone, n=30).

Results: The risk of 28-day mortality was reduced for the high-dose vitamin C versus the standard therapy group (HR=0.14, 95% CI, 0.03-0.72). Oxygen support status was improved more with high-dose vitamin C than standard therapy (63.9% vs 36.1%). No safety events were associated with high-dose vitamin C therapy.

Conclusion: High-dose vitamin C may reduce the mortality and improve oxygen support status in patients with Coronavirus disease 2019 without adverse events.

## INTRODUCTION

Coronavirus disease 2019 (COVID-19) has spread rapidly worldwide [1–5]. As of July 2, 2020, 10,533,779 cases were reported and COVID-19 caused 512,842 death worldwide according to data from the World Health Organization (WHO). More than 50,000 individuals were critically ill [6]. Severe and critically ill patients often have dyspnea and/or hypoxemia and can even rapidly progress to acute respiratory distress syndrome (ARDS), septic shock, and multiple organ failure, resulting in high mortality. The mortality rate ranges from 4% to 28% [7–12].

Previous studies have reported clinical characteristics of patients with COVID-19 [7–9]. Studies showed a rapid and massive production of many cytokines called a cytokine “storm” or inflammatory “storm” in confirmed COVID-19 patients [7–9]. The production of oxygen free radicals leads to microvascular endothelial injury and increased permeability of the microvasculature, resulting in increased exudation, which may be important causes of ARDS and multiple organ failure.

Physicians and biologists all over the world have been looking for drugs for COVID-19. However, no effective antiviral therapy or vaccine has been confirmed. Recently, several clinical trials have investigated therapeutic drugs, [13–19] but their effectiveness and safety are still controversial.

Early studies [20, 21] reported that the application of vitamin C in animal models of sepsis could improve capillary circulation, microvascular barrier function and arteriolar reactivity caused by vasoconstrictors. As a tissue antioxidant, vitamin C can effectively remove oxygen free radicals produced by myocardial tissues, macrophages and ischemia-reperfusion tissues. These free radicals are the initiating factors of Keshan disease [22, 23]. Physicians in our hospital successfully used high-dose vitamin C with patients with acute Keshan disease and cardiogenic shock and reduced the mortality from 86% to 5% [24–26]. In recent years, physicians have used vitamin C to treat various serious inflammatory diseases, especially ARDS and sepsis [27]. In addition, the high-dose vitamin C therapy in acute Keshan disease [24–26] and recent studies [28–30] suggested that a early and short course from 3 to 5 days of high-dose vitamin C treatment could blocked the inflammatory reaction effectively. Observational studies suggested that nearly 40% of sepsis patients showed vitamin C deficiency, [27, 31] and the concentration of vitamin C in plasma of patients with early sepsis was inversely correlated with multiple organ dysfunction indicators [32]. A study of 167 patients with sepsis and ARDS suggested that mortality and intensive care unit stay were significantly reduced in the high-dose vitamin C group [28].

The clinical application of high-dose vitamin C is expected to improve the prognosis of patients by the production of powerful antioxidant free radicals and inhibition of vascular inflammatory exudation. Therefore, we explored the outcomes in patients hospitalized for COVID-19 who received high-dose vitamin C or standard therapy to demonstrate the efficiency and safety of high-dose vitamin C.

## RESULTS

### The exclusion of participants

Overall, 84 patients received high-dose vitamin C or standard therapy, but 8 were excluded because they were pregnant (n=2), lactating (n=1), had missing baseline information (n=1), received fewer than 5 days of high-dose vitamin C (n=3), or died within 24 hr (n=1).

### Baseline characteristics

Table 1 shows the baseline characteristics of 76 patients: 46 with high-dose vitamin C and 30 standard therapy alone. A total of 48 (63.2%) patients had a diagnosis of moderate COVID-19, and 28 (36.8%) severe or critical disease. The median age was 61 years (IQR, 52 to 71), and the median duration of symptoms before therapy 12 days (IQR, 8 to 16). No patient received invasive mechanical ventilation at baseline; 40 (52.6%) patients received high-flow oxygen or noninvasive positive pressure ventilation. The two therapy groups did not differ in baseline characteristics including laboratory data (Table 1, Supplementary Table 1).

**Table 1:** Clinical characteristics of patients at baseline.

**Characteristic**	**Total** **(n=76)**	**High-dose VitC** **(n=46)**	**Standard therapy** **(n=30)**	***P* value**
Disease severity — no. (%)				0.609
Moderate	48 (63.2)	28 (60.9)	20 (66.6)	
Severe or critical	28 (36.8)	18 (39.1)	10 (33.4)	
Age, median (IQR) — years	61 (52-71)	63 (54-71)	57 (49-67)	0.239
Male sex — no. (%)	35 (46.1)	21 (45.7)	14 (46.7)	0.931
Smoking history— no. (%)	8 (10.5)	5 (10.9)	3 (10.0)	0.904
Coexisting condition — no. (%)				
Diabetes	15 (19.7)	11 (23.9)	4 (13.3)	0.257
Hypertension	22 (28.9)	16 (34.8)	6 (20.0)	0.165
Coronary heart disease	5 (6.6)	3 (6.5)	2 (6.7)	0.980
Underlying lung disease	6 (7.9)	4 (8.7)	2 (6.7)	0.748
Chronic liver disease	4 (5.3)	3 (6.5)	1 (3.3)	0.543
Chronic kidney disease	2 (2.6)	2 (4.3)	0	0.247
Systolic blood pressure, median (IQR) — mmHg	130 (112-141)	127 (112-139)	132 (121-144)	0.532
Duration of symptoms before therapy, median (IQR) — days	12 (8-16)	13 (8-20)	10 (8-12)	0.180
Oxygen support category — no. (%)				0.921
Low-flow oxygen	36 (47.4)	22 (47.8)	14 (46.7)	
High-flow oxygen or noninvasive positive pressure ventilation	40 (52.6)	24 (52.2)	16 (53.3)	
Treatment				
Antiviral therapy	72 (94.7)	42 (91.3)	30 (100.0)	0.097
Antibiotic therapy	70 (92.1)	43 (93.5)	27 (90.0)	0.583
Corticosteroids	28 (36.8)	15 (32.6)	13 (43.3)	0.343
Gamma globulin	23 (30.3)	13 (28.3)	10 (33.3)	0.638
Statins	14 (18.4)	10 (21.7)	4 (13.3)	0.355

IQR: interquartile range, VitC: vitamin C.

### Mortality after high-dose vitamin C therapy and standard therapy

**Figure 1:**
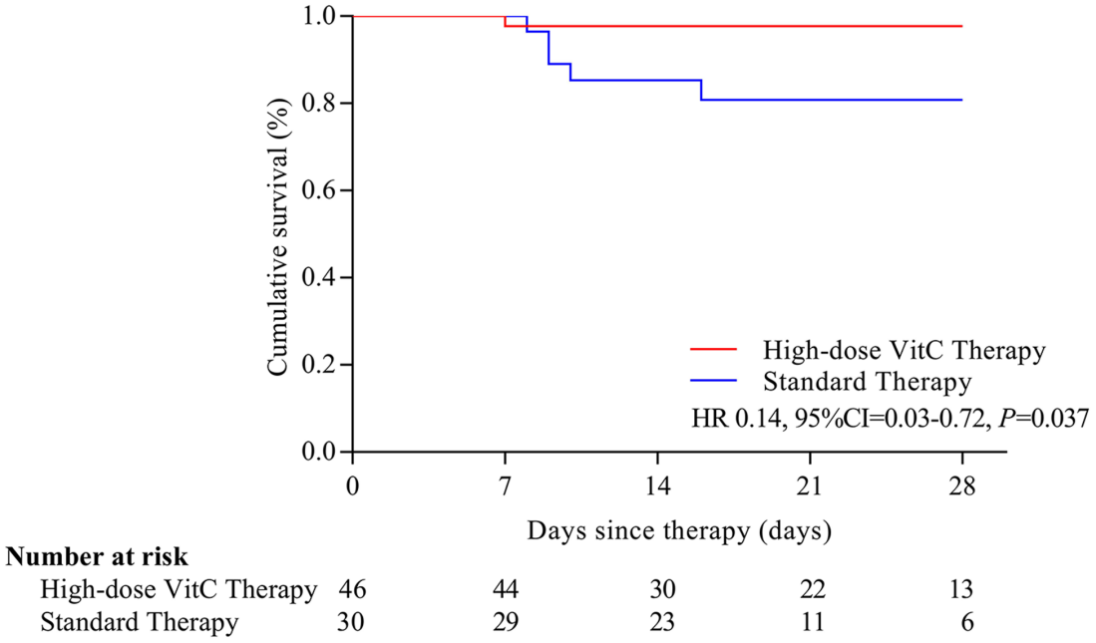
**Overall survival with the two treatments in COVID-19 patients.** The risk of mortality was significantly reduced with high-dose vitamin C than standard therapy (HR=0.14, 95% CI, 0.03-0.72). VitC: vitamin C.

**Figure 2:**
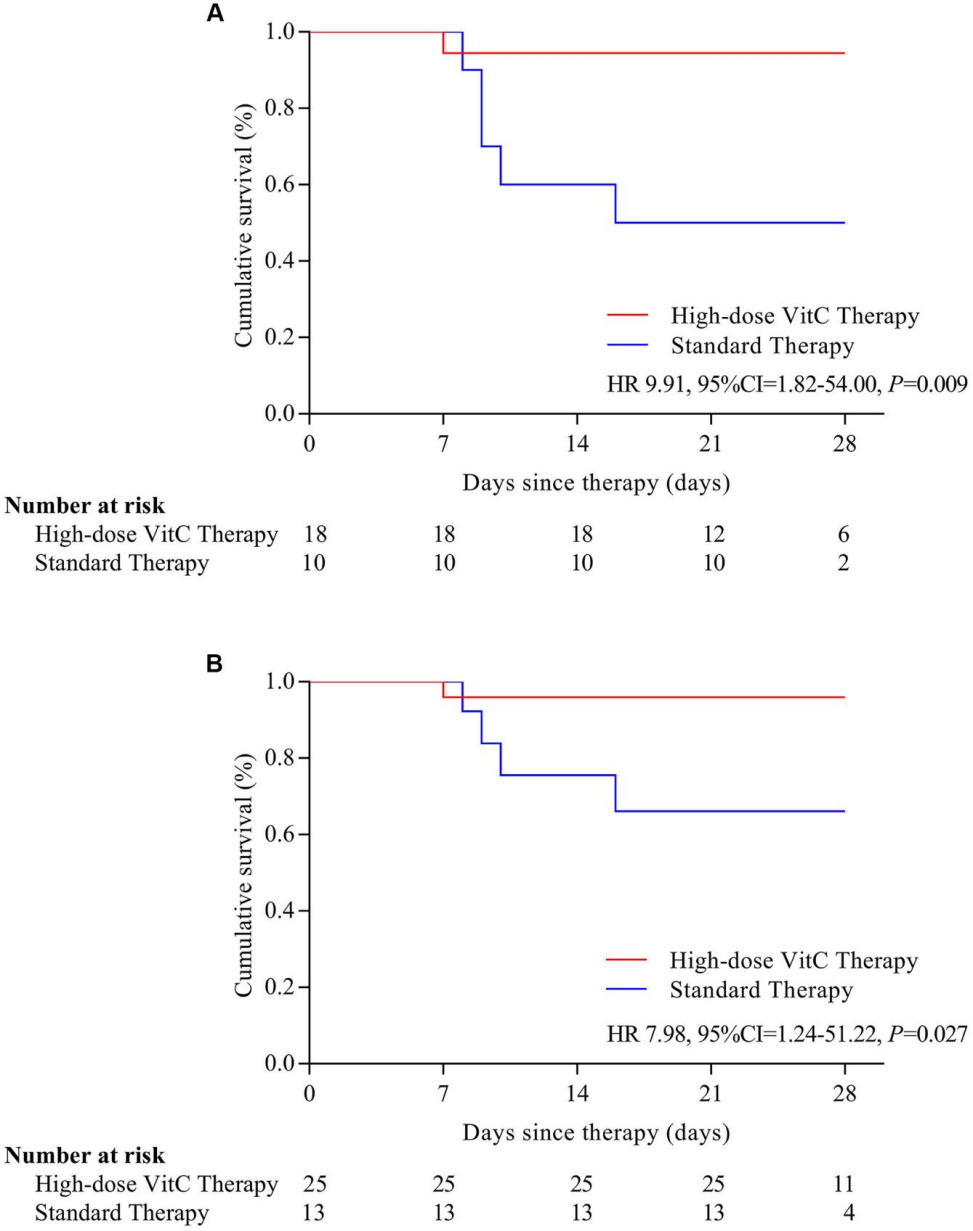
Survival by severe and critical disease (**A**) and age > 60 years old (**B**). Survival is stratified by disease severity at baseline and by age. (**A**) In patients with severe or critical disease, the risk of mortality was lower for patients with high-dose vitamin C than that with standard therapy (HR= 9.91, 95% CI, 1.82-54.00); (**B**) In patients with age > 60, the risk of mortality was lower for patients with high-dose vitamin C than that with standard therapy (HR=7.98, 95% CI, 1.24-51.22). VitC: vitamin C.

Six (7.9%) patients with severe or critical disease died at the end of 28 days; one (16.7%) received high-dose vitamin C, and 5 (83.3%) standard therapy. On Kaplan-Meier analysis, the risk of mortality was significantly reduced with high-dose vitamin C than standard therapy (HR=0.14, 95% CI, 0.03-0.72) (Figure 1). In patients with severe or critical disease and age > 60, the risk of mortality was lower for patients with high-dose vitamin C than that with standard therapy (HR= 9.91, 95% CI, 1.82-54.00; HR=7.98, 95% CI, 1.24-51.22) (Figure 2A, 2B).

### Oxygen support status after high-dose vitamin C therapy and standard therapy

Over a median retrospective time of 18 days (IQR, 10 to 28), 36 (47.4%) patients showed an improvement in oxygen support status, 23 (63.9%) in the high-dose vitamin C group and 13 (36.1%) in the standard therapy group (Table 2). For moderate cases (n=48), oxygen support status was improved for 28 patients, and 17 (60.7%) of them were in the high-dose vitamin C group and 11 (39.3%) in the standard therapy group. For patients with severe or critical disease (n=28), there were 8 patients who showed an improvement, and 6 (75.0%) in the high-dose vitamin C group and 2 (25.0%) in the standard therapy group (Table 2). Moreover, 31 patients were discharged at the end of day 18, 21 (67.7%) in the high-dose vitamin C group, and 10 (32.3%) in the standard therapy group.

**Table 2:** Changes in oxygen-support status after treatment by disease type.

**Oxygen support category**	**Total** **(n=76)**	**High-dose VitC**			**Standard therapy**		
		**Total** **(n=46)**	**Moderate** **(n=28)**	**Severe or critical** **(n=18)**	**Total** **(n=30)**	**Moderate** **(n=20)**	**Severe or critical** **(n=10)**
Low-flow oxygen — no. (%)	14 (18.4)	7 (15.2)	5 (17.9)	2 (11.1)	7 (23.3)	5 (25.0)	2 (20.0)
High-flow oxygen — no. (%)	22 (28.9)	15 (32.6)	7 (25.0)	8 (44.4)	7 (23.3)	6 (30.0)	1 (10.0)
Noninvasive positive pressure ventilation — no. (%)	3 (3.9)	2 (4.3)	0	2 (11.1)	1 (3.3)	0	1 (10.0)
Discharge — no. (%)	31 (40.8)	21 (45.7)	16 (57.1)	5 (27.8)	10 (33.3)	9 (45.0)	1 (10.0)
Death — no. (%)	6 (7.9)	1 (2.2)	0	1 (5.6)	5 (16.7)	0	5 (50.0)
Improvement —no. (%)	36 (47.4)	23 (50.0)	17 (60.7)	6 (33.3)	13 (43.3)	11 (55.0)	2 (20.0)

Percentages of each oxygen support category were calculated with the number of patients at baseline as the denominator.

VitC: vitamin C.

### The subgroups benefit from high-dose vitamin C therapy

In the high-dose vitamin C group, clinical improvement was better for patients ≤ 60 years old than others (HR=0.49, 95%CI, 0.25-0.99, Supplementary Figure 1A). Moreover, clinical improvement was better for patients who received low-flow oxygen (HR=0.41, 95%CI, 0.20-0.84, Supplementary Figure 1B), and those with serum high-sensitivity C-reactive protein (hs-CRP) < 1 mg/L (HR=0.26, 95%CI, 0.07-0.94, Supplementary Figure 1C) than their counterparts.

### Changes in biomarkers of inflammation after high-dose vitamin C therapy and standard therapy

**Figure 3:**
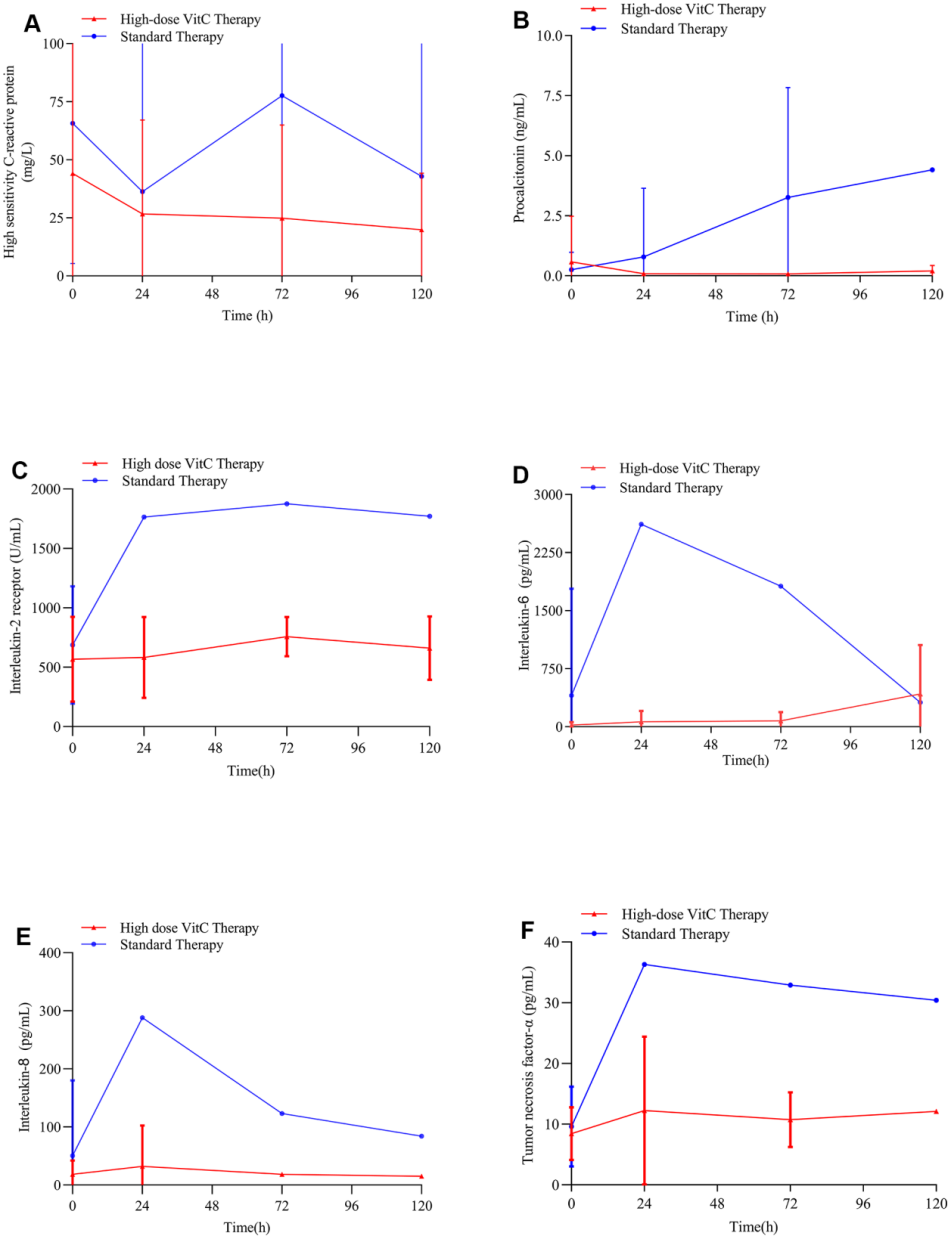
**Changes in hs-CRP and PCT levels with therapy.** (**A**) High-dose vitamin C reduced serum hs-CRP levels in COVID-19 patients; (**B**) High-dose vitamin C reduced serum PCT levels in COVID-19 patients; (**C**) High-dose vitamin C did not affect the serum IL-2R levels in COVID-19 patients remarkably; (**D**) High-dose vitamin C did not affect the serum IL-6 levels in COVID-19 patients remarkably; (**E**) High-dose vitamin C reduced serum IL-8 levels in COVID-19 patients; (**F**) High-dose vitamin C did not affect the serum TNF-α in COVID-19 patients remarkably. VitC: vitamin C; hs-CRP: high-sensitivity C-reactive protein; PCT: procalcitonin; IL-2R: interleukin-2 receptor; IL-6: interleukin-6; IL-8: interleukin-8; TNF-α: tumor necrosis factor-α levels.

As compared with standard therapy, high-dose vitamin C reduced serum hs-CRP, procalcitonin (PCT) and interleukin-8 (IL-8) levels (Figure 3A, 3B, 3E). The serum interleukin-2 receptor (IL-2R), interleukin-6 (IL-6), and tumor necrosis factor-α (TNF-α) levels were not affected remarkably in the high-dose vitamin C group (Figure 3C, 3D, 3F).

### Safety

In total, 19 (41.3%) patients in the high-dose vitamin C group, and 18 (60.0%) in the standard therapy group showed adverse events (Table 3). Thrombocytopenia and increased total bilirubin events were common in the 2 groups. However, the incidence was lower in the high-dose vitamin C than the standard therapy group (8.7% vs 13.3%, 13.0% vs 30.0%). 6 (7.9%) patients showed serious adverse events (respiratory failure or ARDS, shock and sepsis): 1 received high-dose vitamin C and 5 standard therapy. Moreover, respiratory failure or ARDS were more common in the standard therapy than the high-dose vitamin C group.

**Table 3:** Summary of adverse events.

**Event**	**Total** **(n=76)**	**High-dose VitC** **(n=46)**	**Standard therapy** **(n=30)**
Any adverse event	37 (48.7)	19 (41.3)	18 (60.0)
Lymphopenia	7 (9.2)	3 (6.5)	4 (13.3)
Leukopenia	1 (1.3)	1 (2.2)	0
Thrombocytopenia	8 (10.5)	4 (8.7)	4 (13.3)
Increased aspartate aminotransferase activity	5 (6.6)	3 (6.5)	2 (6.7)
Increased alanine aminotransferase activity	3 (3.9)	2 (4.3)	1 (3.3)
Increased total bilirubin level	15 (19.7)	6 (13.0)	9 (30.0)
Increased serum creatinine level	6 (7.9)	3 (6.5)	3 (10.0)
Increased creatine kinase isoenzyme-MB activity	2 (2.6)	1 (2.2)	1 (3.3)
Increased high sensitivity-cardiac troponin I level	5 (6.6)	3 (6.5)	2 (6.7)
Increased N-terminal pro-B-type natriuretic peptide level	5 (6.6)	3 (6.5)	2 (6.7)
Serious adverse event	6 (7.9)	1 (2.2)	5 (16.7)
Respiratory failure or ARDS	5 (6.6)	1 (2.2)	4 (13.3)
Shock	2 (2.6)	1 (2.2)	1 (3.3)
Sepsis	2 (2.6)	1 (2.2)	1 (3.3)

Data are n (%). Adverse events that occurred in more than 1 patient through follow-up. Some patients had more than one adverse event. All deaths were caused by respiratory failure. ARDS: acute respiratory distress syndrome; VitC: vitamin C.

## DISCUSSION

The world is currently facing the threat of the COVID-19 pandemic caused by SARS-CoV-2 infection. This epidemic continues to spread, and there are no vaccines or specific drugs approved or used to prevent or treat COVID-19. A large number of studies have confirmed that high-dose vitamin C can benefit patients with lung injury caused by various inflammatory diseases, especially ARDS and sepsis [27, 30, 33, 34]. This retrospective cohort study analyzed the efficiency and safety of high-dose vitamin C in patients with COVID-19. High-dose vitamin C could decrease mortality and improve the oxygen support status of COVID-19 patients.

Additionally, high-dose vitamin C remarkably reduced serum hs-CRP and PCT levels in COVID-19 patients (Figure 3A, 3B). CRP and PCT are acute-phase inflammatory proteins and related to the severity of body systematic infection. High CRP and PCT levels are associated with organ failure and increased mortality in patients admitted to intensive care units [35, 36]. A study [37] reported lower mortality in COVID-19 patients with reduced CRP level than persistently high CRP level. Jensen and colleagues [35] also found high PCT level as an early independent predictor of mortality for patients admitted to an intensive care unit. Recent reports suggested that COVID-19 patients had high levels of hs-CRP and PCT [36, 38]. Vitamin C can directly reduce the production of reactive oxygen species, maintain endothelial barrier function and vasodilation, and downregulate the expression of proinflammatory modulators [39, 40]. Moreover, high-dose vitamin C reduced serum IL-8 level compared with the standard therapy in COVID-19 patients (Figure 3E). The synergy of cytokines such as TNF-α, interleukin-1 (IL-1), IL-6, and IL-8 can regulate the inflammatory cascade. Studies have shown that vitamin C reduced the production of chemokine such as IL-8, thereby reducing the inflammatory changes of lung injury caused by sepsis, and this reaction was associated with significantly lower mortality in critically ill patients with severe pneumonia [30, 34]. Evidence has suggested that cytokine storms such as higher concentrations of IL-6, IL-8 and interleukin-10 (IL-10) were related to the prognosis of COVID-19 patients [41]. Treatment with high-dose vitamin C is associated with reduced hs-CRP, PCT, and IL-8 levels and can reduce inflammation and thus attenuate lung and systemic inflammation [30, 33, 34]. Also, our COVID-19 patients showed improved oxygen support status, which may lead to reduced mortality.

Epidemiology results showed that patients with severe and critical disease have relatively high mortality, and no effective treatment is available to significantly reduce mortality [42, 43]. Our study found that high-dose vitamin C could significantly decrease mortality with COVID-19 as compared with standard therapy, so vitamin C may be an effective drug for COVID-19, especially for severe and critical cases.

Additionally, we found better clinical improvement with high-dose vitamin C therapy than standard therapy for patients ≤ 60 years old and with low-flow oxygen, and serum hs-CRP level < 1 mg/L. Thus, the use of high-dose vitamin C may have a certain subgroup selectivity, although the clinical improvement between high-dose vitamin C and standard therapy did not significantly differ (Supplementary Figure 2). Body weight is an important clinical indicator for many diseases. While many previous studies [11, 44, 45] have confirmed that the body weight was not associated with the prognosis of patients with COVID-19. In addition, the effect of high-dose vitamin C on anti-oxidation and reducing inflammatory reaction did not show significant correlation with body weight based on recent studies [30, 46]. Although we did not record the body weight of patients in baseline, the body weight might not affect the results of our study.

The incidence of adverse events was lower with high-dose vitamin C than standard therapy, without new adverse events, which suggests that high-dose vitamin C may be safe. Moreover, many studies have confirmed the safety of high-dose vitamin C. A study of high-dose vitamin C treatment in patients with severe sepsis and ARDS found no adverse events [31, 43]. Recent studies [8, 47, 48] reported that some patients with COVID-19 had lymphopenia, leukopenia, thrombocytopenia, liver function abnormality, abnormal myocardial zymography findings, and renal impairment. Therefore, the adverse events in our study were not associated with high-dose vitamin C therapy.

Physicians are exploring various drugs for COVID-19. Remdesivir, [13, 14] hydroxychloroquine/chloroquine [15–17] and corticosteroids [49] have been explored. Although most achieved a certain effect, they were accompanied by various adverse events such as arrhythmia, [18, 19] immunosuppressive effects, [49] liver and kidney damage, [13] and acute respiratory failure [13, 14]. Generally, serious adverse events have a great impact on the treatment effect, prognosis and quality of life of patients. High-dose vitamin C with an effective role and without adverse events might be a promising therapy for COVID-19.

This study has several limitations. First, although we did not observe differences at baseline between the two treatment groups, potential bias exists in this retrospective cohort study. For instance, the treatment patients received and the specific protocols of standard therapy were not controlled. Second, the results are less persuasive owing to the small sample size. Third, we did not collect data on plasma vitamin C level to confirm whether the plasma level is a predictor in patients with COVID-19.

## CONCLUSIONS

In summary, high-dose vitamin C may reduce inflammatory reaction, improve oxygen support status and reduce mortality in COVID-19 patients, without adverse events. Also, it may be effective for certain subgroups with severe and critical disease and older patients. High-dose vitamin C may be a promising therapy for COVID-19.

## MATERIALS AND METHODS

### Study design and participants

This was a retrospective cohort study of in-hospital patients with COVID-19 from January 31, 2020 to March 28, 2020 diagnosed and treated by our medical group in Tongji Hospital, Tongji Medical College, Huazhong University of Science and Technology. This study was based on the approved guidelines for COVID-19 [50]. Informed consent was waived by the board due to the retrospective nature of the study.

Patients with COVID-19 were diagnosed according to Diagnosis and Treatment of Pneumonia Infected by Novel Coronavirus issued by the National Health Commission of China [51]. We excluded patients who were younger than 18 years, allergic to vitamin C, died within 24 hr after admission, or were pregnant and/or lactating. Other patients were classified into two groups: high-dose vitamin C therapy and standard therapy. Patients were informed of the rationality of the treatment plan and potential side effects. Only patients with consent were treated. Patients in the high-dose vitamin C therapy group received standard therapy [51] as well as a 5-day course of a loading dose of 6g added to 5% glucose solution for intravenous high-dose vitamin C infusion lasting over 60 min per 12 hr on the first day, plus 6g added to 5% glucose solution for intravenous high-dose vitamin C infusion lasting over 60 min per day for the following 4 days. Patients receiving standard therapy alone were included in the standard therapy group. The standard therapy was based on the Diagnosis and Treatment Protocol for Novel Coronavirus Pneumonia issued by the National Health Commission of China [51]. As suggested in the guideline, standard therapy included daily monitoring, routine laboratory tests (blood count, urea, creatinine, liver enzymes, and other related biomarkers), effective respiratory therapy (nasal cannula oxygen therapy, mask oxygen therapy or high-flow nasal oxygen therapy, if necessary, noninvasive ventilation), and surveillance of vital parameters according to the patient's condition. The addition of other therapies such as antibiotics, corticosteroids, immunomodulators and other antivirals (e.g., Lopinavir/Ritonavir, Ribavirin) according to the assessment of the physicians were also included in the standard therapy.

This study lasted from the time of hospital admission to discharge or death in hospital occurred. The hospital electronic medical records included the data of all patients from admission to discharge or death in hospital, the data during treatment with high-dose vitamin C was also included. In addition, the 28-day death of patients was recorded.

### Outcomes

The primary outcome was 28-day mortality and clinical improvement. The secondary outcome was change in oxygen support status after treatment. Clinical improvement was defined as a decrease of at least 2 points from baseline to day 28 or discharge according to the seven-category ordinal scale. The ordinal scale is based on the endpoints in patients with severe influenza, [51–53] which consists of 1) no hospitalization with the resumption of normal activities; 2) no hospitalization but unable to resume normal activities; 3) hospitalization but not requiring supplemental oxygen; 4) hospitalization and requiring supplemental oxygen; 5) hospitalization and requiring nasal high-flow oxygen therapy and/or non-invasive mechanical ventilation; 6) hospitalization and requiring extracorporeal membrane oxygenation and/or invasive mechanical ventilation; and 7) death.

### Statistical analysis

Information for patients including clinical characteristics, laboratory data and outcomes were collected from medical records. Continuous variables for baseline characteristics are described with the median (interquartile range [IQR]) and categorical variables with frequency (percentage). Continuous variables were analyzed using Student’s *t* or Mann-Whitney *U* tests and categorical variables were analyzed with chi-squared or Fisher’ s exact tests. Clinical improvement and all-cause mortality were analyzed by Kaplan-Meier analysis with a log-rank test. Hazard ratios and 95% confidence intervals (CIs) were estimated. Data were analyzed by using SPSS 20.0. *P*<0.05 was considered statistically significant.

## Supplementary Material

Supplementary Figures

Supplementary Table 1
