# Supplementary material for: The efficiency and safety of high-dose vitamin C in patients with COVID-19: a retrospective cohort study
Source: Aging (Albany NY). 2021 Feb 26;13(5):7020–34. doi: 10.18632/aging.202557 (PMC7993712; doi:10.18632/aging.202557)
Supplement: Supplementary Figures [file aging-13-202557-s001.pdf]

SUPPLEMENTARY FIGURES

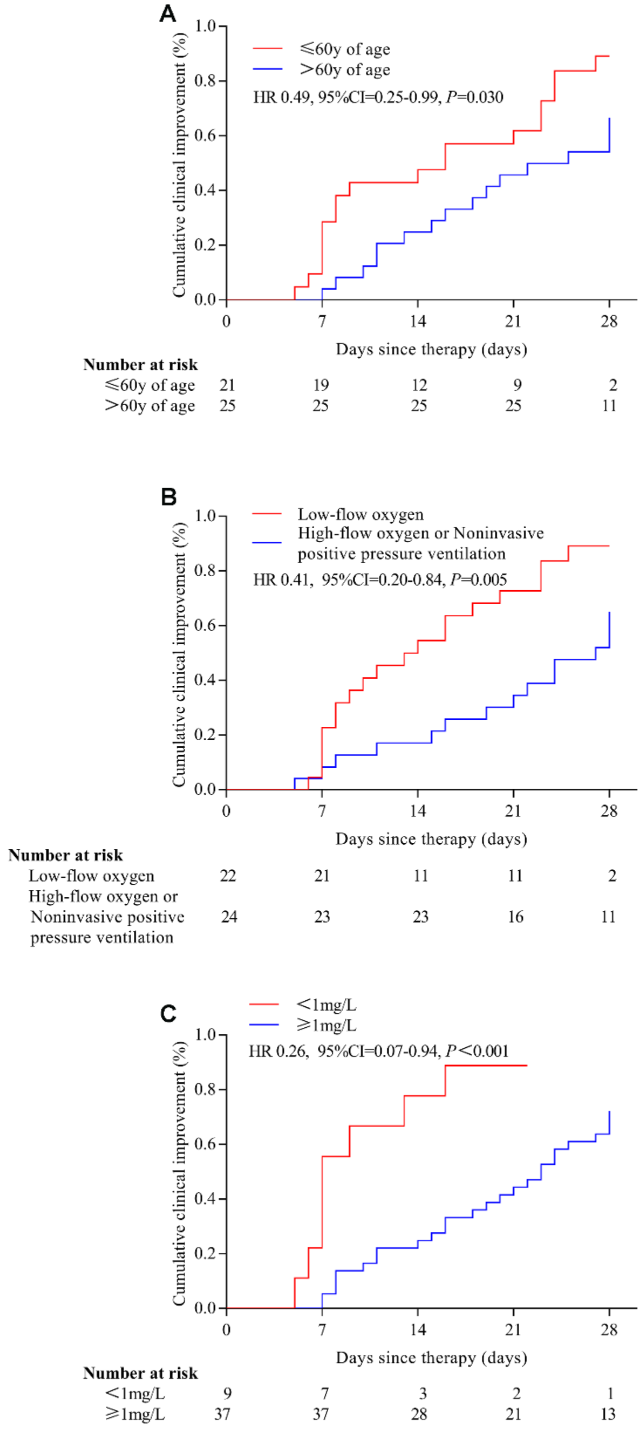

Supplementary Figure 1. Cumulative incidence of clinical improvement in high-dose vitamin C therapy group. (**A**) In the high-dose vitamin C group, clinical improvement was better for patients ≤ 60 years old than others (HR=0.49, 95%CI, 0.25-0.99); (**B**) In the high-dose vitamin C group, clinical improvement was better for patients who received low-flow oxygen (HR=0.41, 95%CI, 0.20-0.84); (**C**) In the high-dose vitamin C group, clinical improvement was better for those with serum hs-CRP < 1 mg/L (HR=0.26, 95%CI, 0.07-0.94). Hs-CRP: high-sensitivity C-reactive protein.

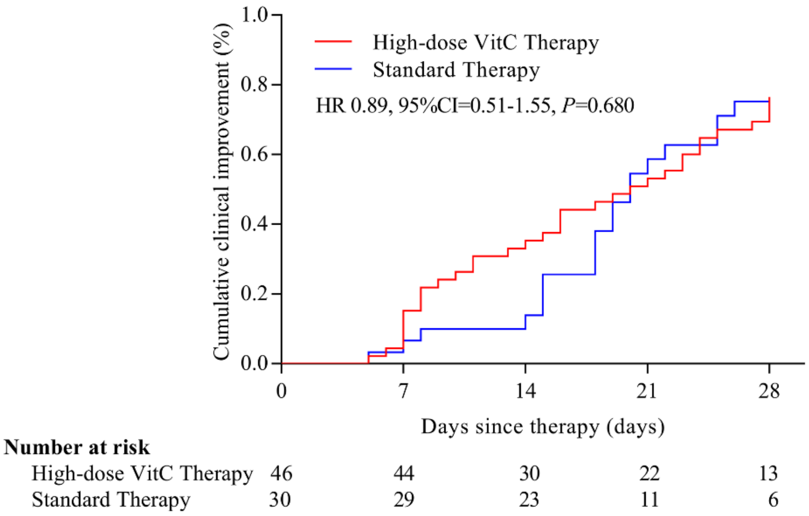

Supplementary Figure 2. Overall cumulative incidence of clinical improvement. The clinical improvement between high-dose vitamin C and standard therapy did not significantly differ (HR=0.89, 95%CI, 0.51-1.55). VitC: vitamin C.
