# Supplementary material for: The efficiency and safety of high-dose vitamin C in patients with COVID-19: a retrospective cohort study
Source: Aging (Albany NY). 2021 Feb 26;13(5):7020–34. doi: 10.18632/aging.202557 (PMC7993712; doi:10.18632/aging.202557)
Supplement: Supplementary Table 1 [file aging-13-202557-s002.pdf]

SUPPLEMENTARY TABLE

Supplementary Table 1. Laboratory data for patients at baseline.

Characteristic	Total (n=76)	High-dose VitC (n=46)	Standard therapy (n=30)	P value
White-cell count (×10−9/L) — median (IQR)	6.7 (4.5-9.1)	6.9 (5.0-9.8)	6.2 (4.4-8.5)	0.276
Lymphocyte count (×10−9/L) — median (IQR)	0.9 (0.7-1.4)	1.0 (0.8-1.6)	0.9 (0.6-1.3)	0.224
Platelet count (×10−9/L) — median (IQR)	210 (168-275)	222 (172-283)	186 (152-231)	0.115
Alanine aminotransferase (U/L) — median (IQR)	22 (14-37)	21 (14-37)	25 (15-38)	0.595
Aspartate aminotransferase (U/L) — median (IQR)	28 (20-43)	26 (17-37)	34 (23-49)	0.059
Serum creatinine (μmol/L) — median (IQR)	67 (60-77)	66 (61-72)	70 (56-83)	0.431
High sensitivity-cardiac troponin I (pg/mL) — median (IQR)	3.5 (2.0-13.6)	3.2 (1.9-15.3)	4.3 (2.3-10.2)	0.765
N-terminal pro-B-type natriuretic peptide (pg/mL) — median (IQR)	113 (47-353)	113 (54-675)	105 (30-217)	0.258
Creatine Kinase Isoenzyme-MB (ng/mL) — median (IQR)	0.9 (0.4-2.2)	0.7 (0.4-2.0)	1.3 (0.3-2.6)	0.754
Lactate dehydrogenase (U/L) — median (IQR)	261 (206-365)	241 (195-356)	309 (229-385)	0.138
High sensitivity C-reactive protein (mg/L) — median (IQR)	9.7 (1.6-76.6)	53.9 (10.0-115.8)	18.7 (3.0-84.2)	0.129
Procalcitonin (ng/mL) — median (IQR)	0.04 (0.02-0.17)	0.07 (0.03-0.11)	0.05 (0.03-0.12)	0.403
Interleukin-2 receptor (U/mL) — median (IQR)	490 (241-899)	630 (356-793)	508 (281-853)	0.353
Interleukin-6 (pg/mL) — median (IQR)	4.70 (1.93-20.02)	3.70(1.78-37.79)	4.69 (1.94-23.49)	0.338
Interleukin-8 (pg/mL) — median (IQR)	11.6 (6.8-20.4)	10.3 (5.8-26.5)	11.3 (6.4-20.4)	0.414
Tumor necrosis factor-α (pg/mL) — median (IQR)	7.3 (5.6-10.1)	8.1 (5.2-10.7)	7.7 (5.6-10.3)	0.472
pO2 (mmHg) — median (IQR)	126.7 (124.3-129.6)	126.7 (124.0-130.9)	126.7 (124.6-128.3)	0.577
pCO2 (mmHg) — median (IQR)	40.1 (39.2-41.0)	40.0 (39.2-41.2)	40.3 (39.3-40.9)	0.992
SO2% (%) — median (IQR)	91.2 (88.7-95.5)	92.9 (88.0-96.0)	90.8 (89.0-92.9)	0.414
TCO2 (mmol/L) — median (IQR)	28.2 (24.5-31.7)	27.9 (23.8-30.6)	29.4 (26.3-32.3)	0.122

IQR: interquartile range; VitC: vitamin C.
